# The Sp1-Responsive microRNA-15b Negatively Regulates Rhabdovirus-Triggered Innate Immune Responses in Lower Vertebrates by Targeting TBK1

**DOI:** 10.3389/fimmu.2020.625828

**Published:** 2021-01-27

**Authors:** Renjie Chang, Qing Chu, Weiwei Zheng, Lei Zhang, Tianjun Xu

**Affiliations:** ^1^ Laboratory of Fish Molecular Immunology, College of Fisheries and Life Science, Shanghai Ocean University, Shanghai, China; ^2^ Key Laboratory of Exploration and Utilization of Aquatic Genetic Resources (Shanghai Ocean University), Ministry of Education, Shanghai, China; ^3^ Laboratory of Marine Biology and Biotechnology, Qingdao National Laboratory for Marine Science and Technology, Qingdao, China; ^4^ National Pathogen Collection Center for Aquatic Animals, Shanghai Ocean University, Shanghai, China

**Keywords:** microRNA, TBK1, regulation, antiviral response, fish

## Abstract

As is known to all, the production of type I interferon (IFN) plays pivotal roles in host innate antiviral immunity, and its moderate production play a positive role in promoting the activation of host innate antiviral immune response. However, the virus will establish a persistent infection model by interfering with the production of IFN, thereby evading the organism inherent antiviral immune response. Therefore, it is of great necessity to research the underlying regulatory mechanisms of type I IFN appropriate production under viral invasion. In this study, we report that a Sp1–responsive miR-15b plays a negative role in siniperca chuatsi rhabdovirus (SCRV)-triggered antiviral response in teleost fish. We found that SCRV could dramatically upregulate miiuy croaker miR-15b expression. Enhanced miR-15b could negatively regulate SCRV-triggered antiviral genes and inflammatory cytokines production by targeting TANK-binding kinase 1 (TBK1), thereby accelerating viral replication. Importantly, we found that miR-15b feedback regulates antiviral innate immune response through NF-κB and IRF3 signaling pathways. These findings highlight that miR-15b plays a crucial role in regulating virus–host interactions, which outlines a new regulation mechanism of fish’s innate immune responses.

## Introduction

Effective detecting of invading viral pathogens by the host innate immune system is essential for the subsequent developing of antiviral innate and adaptive immune responses, which are necessary for clearance of the viruses. Viruses recognition is conducted through different classes of pattern recognition receptors (PRRs), such as Toll-like receptors, retinoic acid-inducible gene (RIG)-I-like receptors (RLRs), and nucleic acid sensor (1, 2). Upon viral infection, these PRRs recruit different key adaptor protein, such as TIR domain-containing adapter-inducing interferon-β (TRIF), mitochondria antiviral-signaling protein (MAVS), or stimulator of interferon genes (STING), which then triggers the activation of TANK-binding kinase 1 (TBK1). Activated TBK1 lead to the activation of interferon-regulatory factor 3 (IRF3) and IRF7 to induce the production of type I interferon (IFN), IFN-stimulated genes (ISGs) and inflammatory cytokines, which are important for elimination of the invading pathogens (3).

TBK1 belongs to a serine/threonine protein kinase of the IKK kinase family, and functions as an essential player in autophagy and mitophagy (4, 5), the insulin signaling pathway (6), and innate inflammatory responses (7, 8). In mammals, the IKK-related kinases TBK1 and IKKϵ function as pivotal components of the virus-activated kinase complex that subsequently phosphorylate and activate IRF3 to induce type I IFN production. Studies have shown that TBK1 and IKKϵ function as key participants in the NF-κB signaling pathway (9). In lower vertebrates, TBK1 have been reported in several teleost species, such as zebrafish, large yellow croaker, Atlantic cod, and grass carp (10–13). Furthermore, growing studies have shown that overexpression of fish TBK1 can significantly inhibit viral replication (10, 13). Overexpression of TBK1 can significantly activate zebrafish IFNI and IFN3 promoters, which can effectively inhibit SVCV infection (10). The mRNA expression of grass carp *Ctenopharyngodon* idella (CiTBK1) was up-regulated and thus mediated the IFN-I signaling pathway after challenged with grass carp reovirus (GCRV) *in vivo* and *in vitro*. And the GCRV production is suppressed in CiTBK1 overexpressing cells, which indicated that CiTBK1 enhanced antiviral immune responses (13). Additionally, fish TBK1 have also been revealed to function as targeted protein of viruses, which could suppress the host type I IFN responses and thus accelerate viral replication (14). In grass carp, TBK1 phosphorylates the Spring viremia of carp virus (SVCV) P protein, reduces the processes of IFN regulatory factor 3 (IRF3) phosphorylation, and inhibits the production of IFN, which in turn promotes viral replication. As a pivotal kinase, only by strictly regulating the activity of TBK1 can the host maintain immune balance. Thus, there are growing evidences to indicate that TBK1 activity can be regulated by many molecules in sets of different ways, including phosphorylation, ubiquitination, kinase activity modulation and prevention of functional TBK1-containing complexes formation (15).

Recently, an increasing number of studies has indicated that host miRNAs function as regulator participated in multiple biological processes (16). MicroRNAs (miRNAs) are small noncoding RNAs (approximately 19–23 nucleotide) that bind to seed sequence of the 3′-untranslated regions (3′-UTRs) from gene transcripts, and it could lead to degradation of the target transcript or inhibition of protein translation through acting as transcriptional suppressor (16, 17). Accumulating evidences have shown that miRNAs play a crucial role in innate antiviral responses. For example, upon FMDV infection, miR-4334-5p regulates interferon (IFN) pathway by targeting ID1 (18). JEV exploits the miRNA regulatory mechanism that miR-155 inhibits the expression of IL-6 and TNF-α by targeting pellino 1 (PELI 1) to evade the host immunity (19). More recently, the function of miRNAs in teleost fish have drawn more and more attention. For instance, miR-3570 in miiuy croaker have been reported to suppress type I IFN and inflammatory cytokine expression by targeting MAVS, finally accelerating virus replication and assisting virus escape (20). Inducible miRNA miR-210 both could regulate RNA virus-triggered antiviral genes and inflammatory cytokine production through NF-κB and IRF3 signaling pathways in teleost fish (21). However, studies on the correlation between miRNA and TBK1 in teleost fish are still unknown.

As a representative group of lower vertebrates, fishes including zebrafish and large yellow croaker are excellent models in immunological research. The innate immune system of teleost fish provides an important basis for its disease resistance (22). Miiuy croaker (*Miichthys miiuy*) as an excellent fish model for extensive researching the innate immune response of fish (23–25), In the present study, the function of miRNAs in host innate antiviral responses and its relationship with TBK1 has been examined in miiuy croaker upon the infection of siniperca chuatsi rhabdovirus (SCRV). As a highly infectious RNA virus, studies have shown that SCRV has seriously threatened the farming of mandarin fish (26). We found that miiuy croaker miR-15b is significantly enhanced following SCRV infection. Furthermore, we found that Sp1 could bind to the miR-15b promoter region and activate its transcription. Upregulated miR-15b subsequently represses the production of SCRV-triggered type I IFN, ISGs, and inflammatory cytokine production by targeting the TBK1, thereby avoiding excessive immunity. It is worth noting that fish miR-15b could modulate antiviral innate immune response through NF-κB and IRF3 signaling pathways. These findings suggest that Sp1–responsive miR-15b play critical roles in the inhibition of an excessive production of type I IFN, which will rich the regulatory networks of miRNA-mediated immune response against pathogen infection in fish species.

## Materials and Methods

### Sample and Challenge

Miiuy croaker (~50 g) was obtained from Zhoushan Fisheries Research Institute, Zhejiang Province, China. Fish was acclimated in aerated seawater tanks at 25°C for six weeks before experiments. The challenge experiments were performed as follows. Briefly, fish was challenged with 100 μl SCRV at a multiplicity of infection (MOI) of 5 or poly(I:C) (InvivoGen, 1mg/ml) through intraperitoneal. As a comparison, 100 μl of physiological saline was used to challenge the individuals. Afterwards, fishes were respectively sacrificed at different time point and the spleen tissues were collected for RNA extraction. All animal experimental procedures were performed in accordance with the National Institutes of Health’s Guide for the Care and Use of Laboratory Animals, and the experimental protocols were approved by the Research Ethics Committee of Shanghai Ocean University (No. SHOU-DW-2018-047).

### Cell Culture

EPC cells were maintained in medium 199 (Invitrogen) supplemented with 10% FBS, 100 U/ml penicillin, and 100 mg/ml streptomycin at 28°C in 5% CO_2_. Miiuy croaker MKC and MIC cells were cultured in L-15 medium (HyClone) supplemented with 15% FBS (Life Technologies). Miiuy croaker macrophages were isolated from head kidney samples as described (27). In brief, tissues were aseptically pushed through a 100-μm nylon mesh to give cell suspension which was then loaded onto 34%/51% Percoll (Pharmacia, USA) density gradient to obtain macrophages. The cells were cultured in L-15 (Hyclone) medium supplemented with 20% FBS and seeded into six-well plates at 26°C in 4% CO_2_.

### Plasmids Construction

To construct the TBK1 3′-UTR reporter vector, the 3′-UTR regions of miiuy croaker TBK1 gene was amplified using PCR and cloned into pmir-GLO luciferase reporter vector (Promega) using *Nhe* I and *Xba* I sites restriction sites. Similarly, the 3′-UTR region of *D. rerio* TBK1 gene or *L. crocea* TBK1 gene was amplified and inserted into *Nhe* I and *Xho* I restriction sites of pmir-GLO vector. The mutant-types of TBK1 3′-UTR reporter were constructed by using Mut Express II Fast Mutagenesis Kit V2 (Vazyme) with specific primers (**Supplementary Table 1**). Moreover, the wild type of miiuy croaker TBK1 3′-UTR or its mutant-type reporter plasmid were inserted into the mVenus-C1 (Invitrogen). The mVenus-C1 vector included the sequence of enhanced GFP. In addition, to construct the pre-miRNA vector, the pre-miR-15b sequence was PCR-amplified and then cloned into pcDNA3.1 vector (Invitrogen). To construct the TBK1 expression plasmid, the full length of CDS region and 3′-UTR of miiuy croaker TBK1 gene were amplified by specific primer pairs with the Flag tag, and cloned into pcDNA3.1 vector (Invitrogen). The luciferase reporter plasmid TSS-2417, TSS-2025, TSS-1303, TSS-979, and TSS-427, which contain the 2417-, 2025-, 1303-, 979-, and 427-bp proximal promoter sequences of miiuy croaker miR-15b, respectively, were constructed by PCR amplification using genomic DNA of miiuy croaker as templates and subsequent cloning into pGL3-basic vector (Promega). To construct the Sp1 expression plasmid, the full length of CDS region of miiuy croaker Sp1 gene were amplified by specific primer pairs with the Flag tag, and cloned into pcDNA3.1 vector (Invitrogen). miR-15b promoter reporter constructs containing mutations for Sp1 were constructed with specific primers (**Supplementary Table 1**). The correct construction of the plasmids was verified by Sanger sequencing and extracted through Endotoxin-Free Plasmid DNA Miniprep Kit (Tiangen).

### miR-15b Target Gene Identification

The miR-15b targets were predicted using Targetscan (28), miRanda (29), and MicroInspector (30) algorithms. Predictions were ranked based on the predicted efficacy of targeting as calculated using the context and scores of the sites.

### miRNA Mimics and Inhibitors

miR-15b mimics (dsRNA oligonucleotides), miR-15b inhibitors (single-stranded oligonucleotides), and control oligonucleotides were ordered from GenePharma (Shanghai, China). The sequences are as follows: miR-15b mimics were 5′-CGAACCAUUAUUUGCUGCUUUA-3′ (sense), 5′-AAGCAGCAAAUAAUGGUUCGUU-3′ (antisense); negative control mimics were 5′-UUCUCCGAACGUGUCACGUTT-3′ (sense), 5′-ACGUGACACGUUCGGAGAATT-3′ (antisense); miR-15b inhibitors were 5′-TAAAGCAGCAAATAATGGTTCG-3′ (chemically modified by 2′-Ome); negative control inhibitors were 5′-CAGUACUUUUGUGUAGUACAA-3′.

### RNA Interference

The TBK1-specific siRNA (si-TBK1) were 5′-GUUCGAGGUCCUGAAGAAATT-3′ (sense) and 5′-UUUCUUCAGGACCUCGAACTT-3′ (antisense). The Sp1-specific siRNA (si-Sp1) were 5′-GAGAGAACGAUUCACCUUCTT-3′ (sense) and 5′-GAAGGUGAAUCGUUCUCUCTT-3′ (antisense). The scrambled control RNA sequences were 5′-UUCUCCGAACGUGUCACGUTT-3′ (sense) and 5′-ACGUGACACGUUCGGAGAATT-3′ (antisense).

### Cell Transfection and Virus Infection

Transient transfection of cells with miRNA mimic, miRNA inhibitor or siRNA was performed in 24-well plates using Lipofectamine^™^ RNAiMAX (Invitrogen), and cells were transfected with DNA plasmids was performed using Lipofectamine™ 3000 (Invitrogen) according to the manufacturer’s instructions. For functional analyses, the expression plasmid (500 ng per well) or empty plasmid (500 ng per well) and miRNA mimics (100 nM), miRNA inhibitor (100 nM) or siRNA (100 nM) were transfected into cells in culture medium and then harvested for further detection. For luciferase experiments, miRNA mimics (100 nM) or miRNA inhibitor (100 nM) and pmir-GLO (500 ng per well) containing the wild or mutated sequence of TBK1 3′UTR were transfected into cells.

Miiuy croaker macrophages were seeded in 12-well plates overnight before stimulation. Cells were washed and infected with SCRV at a multiplicity of infection (MOI) of 5 or poly(I:C), and incubated for the different time as indicated. The cells were also infected with SCRV at a MOI of 0.01, 0.1, 1, and 10. Total RNA was extracted for qRT-PCR analysis

### Dual-Luciferase Reporter Assays

The wild-type or mutant-type of TBK1 3′-UTR luciferase reporter was cotransfected with miR-15b mimics, miR-15b inhibitors, the pre-miR-15b plasmid or their negative controls into HEK293 cells or EPC cells. To determine the functional regulation of miR-15b, EPC cells were cotransfected with reporter genes (20, 21), TBK1 expression plasmid, pRL-TK renilla luciferase plasmid, together with miR-15b mimics, pre-miR-15b plasmid or negative controls for dual-luciferase reporter assays. Afterwards, the cells were lysed for reporter activity testing using the dual-luciferase reporter assay system (Promega). All the luciferase activity values were achieved against the renilla luciferase control. For each experiment, three independent experiments were conducted, and each experiment was done in triplicate.

### Western Blotting

Cellular lysates were generated by using 1×SDS-PAGE loading buffer. Proteins were extracted from cells and measured with the BCA Protein Assay kit (Vazyme), then subjected to SDS-PAGE (10%) gel and transferred to PVDF (Millipore) membranes by semidry blotting (Bio-Rad Trans Blot Turbo System). The membranes were blocked with 5% BSA. Protein was blotted with different antibodies. The antibody against TBK1 was diluted at 1: 400 (BOSTER, BA3138); anti-Flag, and anti-Tubulin monoclonal antibody were diluted at 1: 2,000 (Sigma); and HRP-conjugated anti-rabbit IgG or anti-mouse IgG (Abbkine) at 1: 5,000. The results were the representative of three independent experiments. The immunoreactive proteins were detected by using WesternBright™ ECL (Advansta). The digital imaging was performed with a cold CCD camera.

### RNA Extract and Quantitative Real-Time PCR

The viral RNA in the intracellular and supernatant was extracted by using the Body Fluid Viral DNA/RNA Miniprep Kit (Axygen). Total RNA was isolated with TRIzol Reagent (Invitrogen) and the cDNA was synthesized using the FastQuant RT Kit (Tiangen) which includes DNase treatment of RNA to eliminate genomic contamination. The expression patterns of each gene were performed by using SYBR Premix Ex Taq™ (Takara). The small RNA was extracted by using miRcute miRNA Isolation Kit (Tiangen), and miRcute miRNA FirstStrand cDNA Synthesis Kit (Tiangen) was applied to reverse transcription of miRNAs. The expression analysis of miR-15b was executed by using the miRcute miRNA qPCR Detection Kit (Tiangen). Real-time PCR was performed in an Applied Biosystems^®^ QuantStudio 3 (Thermo Fisher Scientific). GAPDH and 5.8S rRNA were employed as endogenous controls for mRNA and miRNA, respectively as described (21). Primer sequences are displayed in **Supplementary Table 1**.

### Virus Yield Quantification

MKC cells were transfected with oligonucleotides and then infected with SCRV (MOI = 5). A volume of 0.1 ml of the cultural supernatant was then serially diluted on the monolayer of EPC cells, and EPC cells were seeded into 96-well plates 24 h before measurement. The 50% tissue culture infectious dose (TCID_50_) was measured after 72 h. The viral RNA in the intracellular was extracted by using the Body Fluid Viral DNA/RNA Miniprep Kit (Axygen), and SCRV RNA replicates were quantified by qRT-PCR.

### Statistical Analysis

Data are expressed as the mean ± SE from at least three independent triplicated experiments. Student’s t-test was used to evaluate the data. The relative gene expression data was acquired using the 2 ^∆∆CT^ method, and comparisons between groups were analyzed by one-way analysis of variance (ANOVA) followed by Duncan’s multiple comparison tests (31). A value of *p* < 0.05 was considered significant.

## Results

### Viral Infection Enhances the Expression of miR-15b

**Figure 1:**
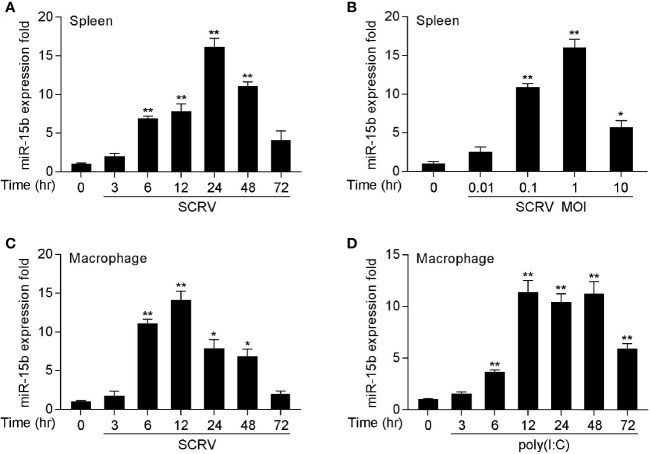
miR-15b is upregulated in miiuy spleen tissues and macrophages following SCRV and poly(I:C) treatment. **(A, B)** The relative expression of miR-15b in spleen samples were measured by qRT-PCR at indicated time after SCRV (MOI = 5) infection **(A)** or various SCRV MOIs **(B)**. **(C)** Expression of macrophage miR-15b was detected by qRT-PCR upon SCRV (MOI=5) **(C)** or poly(I:C) **(D)** infection at different times. And the qRT-PCR analysis was normalized to 5.8S rRNA. Results are standardized to 1 in control samples. All data represented the mean ± SE from three independent triplicated experiments. **p < 0.01; *p < 0.05 versus the controls.

To determine whether miRNAs may be involved in regulating SCRV infection in fish species, we performed a comprehensive miRNA profiling of SCRV-infected miiuy croaker (data not shown). Here, a series of differentially expressed miRNAs following SCRV treatment have been screened out through deep sequencing analysis. Among them, we selected miR-15b which presented significantly upregulated expression after pathogen infection (the log2(Foldchange) of miR-15b was 1.28, and its *p*-value was 0.03). To verify the results obtained by RNA-Seq profiling, miR-15b expression was examined by quantitative real-time PCR (qRT-PCR) at multiple time points after SCRV infection or different kinds of SCRV infection MOIs. In accordance with the RNA-Seq data, the levels of miR-15b in miiuy croaker spleen samples presented the upward trend (**Figure 1A**); this response also showed a dose dependent manner (**Figure 1B**). We also infected miiuy croaker macrophages with SCRV to detect miR-15b expression, and the results showed the expression levels of miR-15b were observably increased in a time-dependent manner (**Figure 1C**). Meanwhile, we examined miR-15b expression by using poly(I:C), an interferon inducer with double stranded RNA. Similar to SCRV infection, poly(I:C) stimulation could also enhance miR-15b expression in macrophages (**Figure 1D**). These data verified that miR-15b expression could be increased by SCRV infection, which indicated that miR-15b potentially participates in regulating the immune responses triggered by virus infection.

### Sp1 Activates miR-15b Transcription

**Figure 2:**
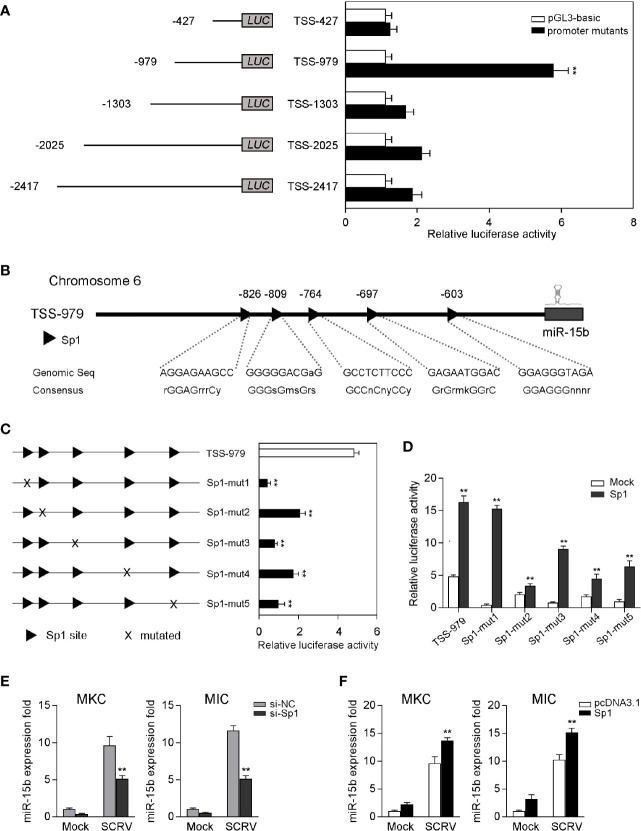
Sp1 sites are essential for the transcriptional initiation of miR-15b promoter. **(A)** The dual-luciferase activity analysis was conducted to screen the fully intact promoter of miR-15b. **(B)** The diagram of transcriptional binding sites. **(C)** Representative results of luciferase activity assays of EPC cells after mutant miR-15b promoter reporter transfection. **(D)** The effect of Sp1 expression construct on the wild-type miR-15b promoter reporters (TSS-979-wt) or the mutated miR-15b promoter reporters (TSS-979-mut) was measured by dual-luciferase assays. Luciferase activities were measured at 24 h post-transfection. All the luciferase activity was normalized to renilla luciferase activity. **(E, F)** The qRT-PCR analysis of Sp1 expression in MKC and MIC cells after transfection with Sp1 siRNA or control siRNA **(E)** and Sp1 expression plasmid or pcDNA3.1 under SCRV stimulation **(F)**. The miR-15b expression levels were normalized to 5.8S rRNA, and results are standardized to 1 in control samples. All data are presented as the means ± SE from at least three independent triplicated experiments. **p < 0.01 versus the controls.

To examine the roles of miR-15b played *via* transcriptional regulation, we obtained the genomic information of miR-15b including the predicted transcription start site through the whole genome query in miiuy croaker (24). To investigate the totally intact promoter of miR-15b, a group of reporter plasmids within different length were constructed from the sequence upstream of the predicted transcription start site (TSS) of miR-15b to detected the promoter luciferase activity. As shown in **Figure 2A**, the luciferase reporter TSS-979 could be capable of producing the highest luciferase activity compared with other reporters. There is a 6.3-fold luciferase activity induced by TSS-979 when compared with the pGL3-basic empty vector, which indicated TSS-979 owning totally intact promoter activity. These data indicated that the complete promoter activity region from positions −979 to −427 is essential, and the negative regulatory element(s) may exit in the promoter region from positions −1303 to −979, and the *cis*-acting elements located in the promoter region coordinate transcriptional initiation of miR-15b.

Afterwards, the transcription factors in the TSS-979 luciferase reporter plasmid related to miR-15b transcription were analyzed by Alibaba (v2.1) software. The sites of the transcription factor Sp1 in the promoter region were predicted (**Figure 2B**). To explore the necessity of these transcription factors, five mutations that either mutated Sp1 site in TSS-979 luciferase reporter was constructed. According to **Figure 2C**, mutation in any Sp1 binding sites on miR-15b promoter led to a markedly decrease of luciferase activity, which suggests that all Sp1 sites are indispensable for miR-15b transcription and that mutation of any one of them attenuates the promoter activity. Further, the gain- and loss-of-function assays were performed to indicate that increased Sp1 expression plasmid induced an increase of the luciferase activity of miR-15b promoter mutations (**Figure 2D**). These results indicated that 5 mutations of Sp1 binding sites weakened the transcriptional activity mediated by Sp1 overexpression, and also indicated the important role for the Sp1 site in the transcription of miR-15b. To examine whether Sp1 activates the expression of miR-15b, we tested the effect of inhibition or overexpression of Sp1 on the level of miR-15b. To this end, we transfected miiuy croaker kidney cells (MKC) and miiuy croaker intestines cells (MIC) with Sp1-specific siRNA (si-Sp1), then treated cells with SCRV. The qRT-PCR results showed that miR-15b expression was downregulated after knockdown of Sp1 (**Figure 2E**). Moreover, we also overexpressed Sp1 in both MKC and MIC cells. As expected, the expression of miR-15b was upregulated after transfection of Sp1 expression plasmid (**Figure 2F**). Collectively, these results suggested that the sequence from positions −979 to −427 is necessary for basal miR-15b promoter activity, meanwhile Sp1 plays an important role in miR-15b transcription.

### miR-15b Participates in Modulating SCRV-Triggered Antiviral Responses

**Figure 3:**
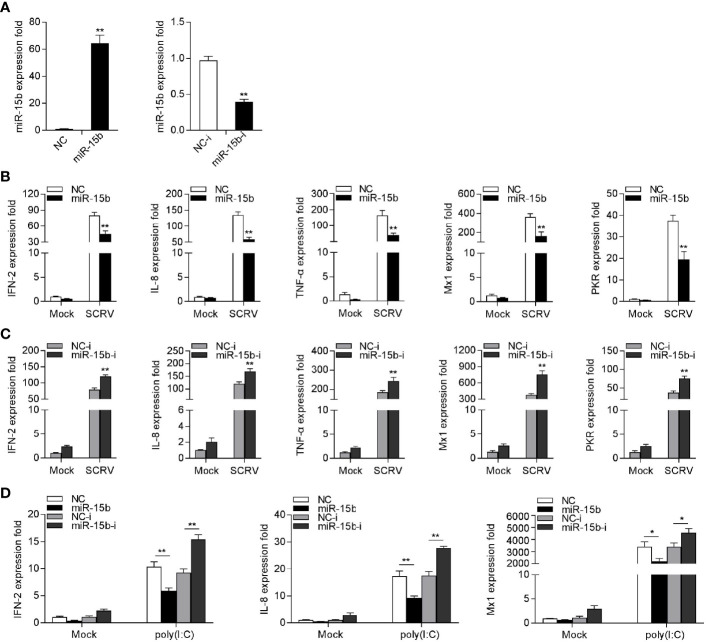
miR-15b suppresses SCRV-triggered type I IFN and inflammatory cytokine expression. **(A)** Analysis of the function of miR-15b mimics and miR-15b inhibitors to miR-15b in MKC cells. miR-15b expression was measured by qRT-PCR and normalized to 5.8S rRNA. **(B)** MKC cells were transfected with NC or miR-15b for 48 h and infected with SCRV for 24 h. Then, qRT-PCR analysis was conducted to measure the expression levels of IFN-2, IL-8, TNF-α, Mx1, and PKR **(C)** MKC cells were transfected with NC-i or miR-15b-i for 48 h, and the cells were treated with SCRV. The expression levels of the indicated genes were analyzed by qRT-PCR. **(D)** MKC cells were transfected with NC, miR-15b, NC-i or miR-15b-i for 48 h. Then, the cells were stimulated with poly(I:C) for 24 h and the expression levels of IFN-2, IL-8, and Mx1 were analyzed by qRT-PCR. From **(B–D)**, all qRT-PCR analysis was normalized to GAPDH. Results are standardized to 1 in control cells. All data are presented as the means ± SE from three independent triplicated experiments. **p < 0.01; *p < 0.05 versus the controls.

To explore the potentially regulatory mechanisms of miR-15b in miiuy croaker antiviral immune response, we measured the effect of overexpression or inhibition of miR-15b on type I IFN and inflammatory cytokines production following SCRV treatment. To this end, the role of synthetic miR-15b mimics and miR-15b inhibitors were used to estimate miR-15b expression level in MKC firstly. miR-15b mimics are synthetic dsRNAs that simulate naturally occurring mature miRNAs, while miR-15b inhibitors are chemically modified antisense ssRNAs that sequester and inhibit intracellular miRNAs. As expected, miR-15b mimics strengthened miR-15b level, but miR-15b inhibitors impaired miR-15b level. Afterwards, the role of overexpression or inhibition of miR-15b in the expression levels of IFN, ISGs, and inflammatory factors were investigated in SCRV-infected MKC cells. The cells were transfected with miR-15b mimics or control mimics, then treated with SCRV stimulation. We can see from **Figure 3B** that forced expression of miR-15b could be able to reduce the expression levels of SCRV-induced antiviral genes, containing IFN-2, IL-8, TNF-α, Mx1, and PKR. In contrast, as shown in **Figure 3C**, the inhibition of endogenous miR-15b showed an upward trend on the expression of these indicated genes compared with transfection of control inhibitors. To further investigate the function of miR-15b in antiviral response, poly(I:C) was used as a stimulus to treat MKC cells, then the expression of antiviral genes was tested. As can be seen from **Figure 3D**, miR-15b mimics significantly decreased the expression levels of IFN-2, IL-8, and Mx1, and the inhibition of miR-15b expression enhanced these genes expression contrary. Taken together, above data demonstrated that miR-15b plays a negative role in regulating a series of RNA virus-induced immune response in MKC cells.

### miR-15b Targets TBK1

**Figure 4:**
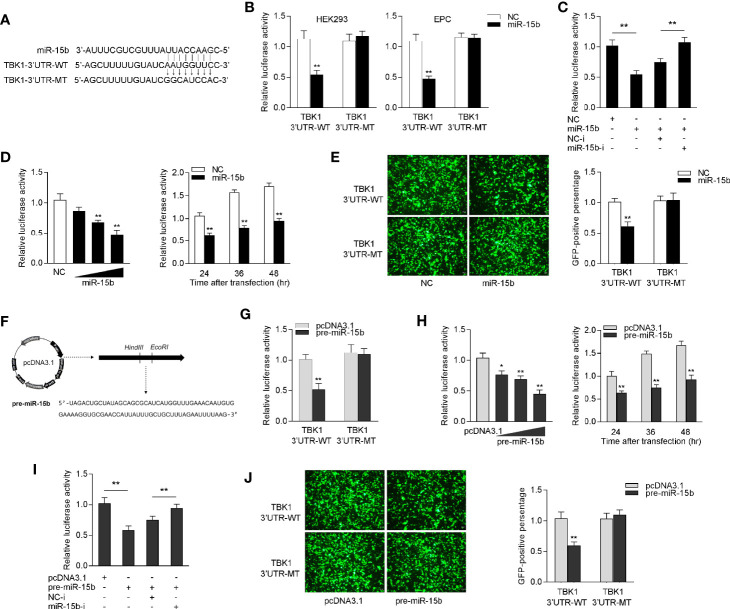
miR-15b targets miiuy croaker TBK1 gene. **(A)** Diagrammatic drawing of miR-15b and its predicted binding sites in TBK1 3′UTR. **(B)** The dual-luciferase reporter assay was applied to detect the regulation of miR-15b mimics on the wild-type or the mutant-type of TBK1 3′-UTR luciferase reporters. **(C)** The luciferase activity was tested to confirm the role of NC, miR-15b, NC-i or miR-15b-i through cotransfection with TBK1 3′-UTR reporters in EPC cells. **(D)** The concentrate gradient (left panel) and time gradient (right panel) experiments were conducted for miR-15b transfection. For the concentrate gradient experiments, miR-15b (0, 25, 50, and 100 nM) together with negative control (100, 75, 50, and 0 nM) was cotransfected into EPC cells. **(E)** EPC cells were cotransfected with the wild type or mutant type of mVenus-TBK1-3′UTR, together with NC or miR-15b. At 48 h post-transfection, the fluorescence intensity was observed with Leica DMiL8_fluorescence microscope and evaluated by Thermo Scientific Varioskan LUX. Scale bar, 20 μm; original magnification ×10. **(F)** The precursor sequence of miR-15b and its construction process. **(G)** The dual-luciferase reporter assayed the regulation of pre-miR-15b on the wild-type or the mutant-type of TBK1 3′-UTR luciferase reporters. **(H)** The concentrate gradient (left panel) and time gradient (right panel) experiments were conducted for pre-miR-15b transfection. For the concentrate gradient experiments, pre-miR-15b plasmid (0, 100, 200, and 500 ng) along with pcDNA3.1 vector (500, 400, 300, and 0 ng) was cotransfected into EPC cells. **(I)** The luciferase activity was tested to confirm the role of pcDNA3.1, pre-miR-15b, NC-i or miR-15b-i through transfection with TBK1 3′-UTR reporters in EPC cells. **(J)** EPC cells were cotransfected with the wild type or mutant type of mVenus-TBK1-3′UTR, together with pcDNA3.1 or pre-miR-15b. The fluorescence intensity was observed after 48h transfection. All the luciferase activity was normalized to renilla luciferase activity. All data are presented as the means ± SE from at least three independent triplicated experiments. **p < 0.01; *p < 0.05 versus the controls.

To identify the possible target of miR-15b, the miRNA target prediction programs was used to search for potential miR-15b targets, and a total 24 sequences have been predicted as potential miR-15b targets (**Supplementary Table 2**). Among the predicted targets of miR-15b, we paid attention to TBK1, which could be as to a mediator in antiviral immune responses. Through prediction analysis, TBK1 gene harbor a standard target sequence for miR-15b at its 3′-UTR (**Figure 4A**). To assess whether miR-15b directly target TBK1 gene through the seed sequence in the 3′-UTR regions, the luciferase reporter plasmid with the putative TBK1 3′-UTR target sites for miR-15b (TBK1 3′UTR WT) was constructed, as well as mutant type (TBK1 3′UTR MT). Both TBK1 luciferase reporter plasmids together with miR-15b mimics or the control mimics were cotransfected into HEK293 cells or Epithelioma papulosum cyprinid (EPC) cells. According to **Figure 4B**, there is a marked decline in relative luciferase activity when TBK1-3′UTR WT was cotransfected with miR-15b mimics, and this suppression was abolished by deleting part of the perfectly complementary sequences in the TBK1 3′-UTR. Furthermore, the assay from **Figure 4C** was conducted to further confirm the negative regulation mechanism of miR-15b mimics and inhibitors, and the results indicated that the cotransfection of miR-15b inhibitors plays an attenuation role in the inhibited luciferase activity. miR-15b mimics showed inhibitory effect on luciferase activity in a concentration-dependent manner from 24 to 48 h after transfection (**Figure 4D**). In addition, miR-15b mimics have been exhibited to suppress GFP gene expression. To be more precise, only the inhibition effect appeared when the TBK1 3′-UTR (WT) with mVenus-C1 was transfected into EPC cells, and that there is no effect on GFP gene expression within the mutant type constructs (**Figure 4E**).

Considered that miRNA processing system is evolutionary conserved in both invertebrates and vertebrates (32), we constructed the precursor plasmid of miR-15b and verified the role of pre-miR-15b by transfection of it into EPC cells (**Figure 4F**). After transfection of pre-miR-15b plasmid, we found that the luciferase activity was sharply declined by pre-miR-15b overexpression, whereas there is no effect on luciferase activity when transfected with the mutant type constructs in EPC cells (**Figure 4G**). Likewise, the dose- and time-gradient experiments of pre-miR-15b were conducted. As can be seen from **Figure 4H**, the pre-miR-15b plasmid has a dose-dependent inhibitory effect on luciferase activity, and it signally inhibits the luciferase activity after 48 h transfection. When the EPC cells was cotransfected with the luciferase reporter plasmids, the pre-miR-15b plasmid and miR-15b inhibitors, we observed that pre-miR-15b plasmid dramatically reduced luciferase activity, and miR-15b inhibitors could effectively attenuate the inhibition effect create by pre-miR-15b (**Figure 4I**). For further validation, as shown in **Figure 4J**, the results showed that pre-miR-15b plasmid could effectively decrease GFP expression, whereas no change in fluorescence intensity was obtained compared with transfection of the mutant form. Together, above consequences adequately demonstrated that TBK1 has a potential target binding region for miR-15b and miR-15b directly targets TBK1 gene.

### miR-15b Could Posttranscriptional Repress TBK1 Expression

**Figure 5:**
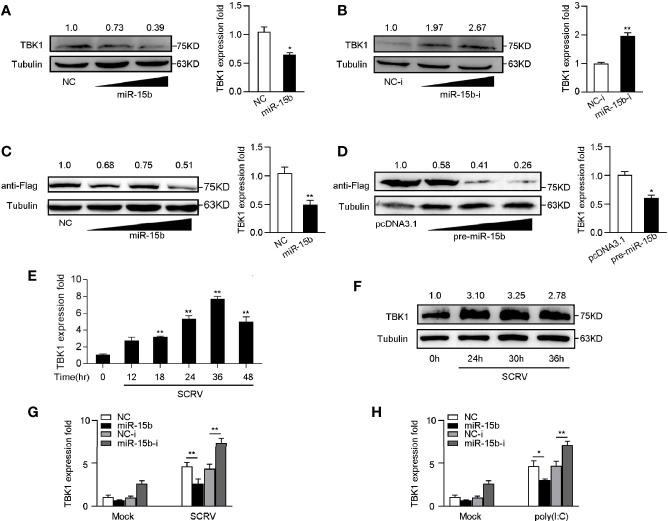
miR-15b negatively regulates the expression of TBK1 at posttranscriptional level. **(A, B)** TBK1 expression was determined by western blotting or qRT-PCR in MKC cells within NC or miR-15b **(A)** and NC-i or miR-15b-i **(B)** treatment, separately. **(C, D)** TBK1 expression level was examined by western blotting or qRT-PCR in EPC cells within NC or miR-15b **(C)** and pcDNA3.1 or pre-miR-15b **(D)** treatment, respectively. **(E, F)** The expression profiles of TBK1 in macrophages were detected by qRT-PCR **(E)** and western blotting **(F)** respectively at indicated time points after SCRV (MOI = 5) infection. **(G, H)** After transfection of NC, miR-15b, NC-i or miR-15b-i for 48 h, MKC cells were treated with SCRV **(G)** or poly(I:C) **(H)** for 24 h. Then qRT-PCR was used to measure TBK1 expression in MKC cells. All the qRT-PCR analysis was normalized to GAPDH. The results are standardized to 1 in control cells. All data are presented as the means ± SE from three independent triplicated experiments. **p < 0.01; *p < 0.05 versus the controls.

To determine whether miR-15b participates in the regulation of TBK1 expression, we examined the endogenous TBK1 levels in MKC cells which is transfected with miR-15b mimics or miR-15b inhibitors. According to **Figures 5A, B**, overexpression of miR-15b obviously suppressed endogenous TBK1 protein and mRNA expression level in a dose-dependent manner, and TBK1 expression could be increased through the miR-15b inhibitors transfection. These results suggested that miR-15b could negatively regulate endogenous TBK1 expression in both protein and mRNA levels. Moreover, to further identify TBK1 is a target of posttranscriptional repression by miR-15b, we constructed TBK1 expression plasmid that contains miiuy croaker TBK1 full-length coding sequence (CDS) region and 3′-untranslated regions (3′-UTR). Then the TBK1 expression plasmid, along with miR-15b mimics or the precursor plasmid were transfected into EPC cells. As expected, miR-15b mimics, as well as pre-miR-15b plasmid, not only obviously inhibited the levels of TBK1 protein, but also decreased the mRNA expression of TBK1 (**Figures 5C, D**). Then, we infected MKC cells with SCRV to detect TBK1 expression, and the results showed the expression levels of TBK1 were observably strengthened in a time-dependent manner (**Figures 5E, F**). Furthermore, to test whether inducible miR-15b decreases the level of TBK1 upon virus treatment, transfection of miR-15b mimics or miR-15b inhibitors into MKC cells was implemented and then treated with SCRV or poly(I:C). As shown in **Figure 5G**, transfection of miR-15b mimics could almost abolish upregulation of TBK1 expression caused by SCRV infection in MKC cells. On the contrary, the induction of miR-15b inhibitors markedly enhanced TBK1 mRNA levels compared with control inhibitors. Meanwhile, a similar regulation of TBK1 expression was found in MKC cells upon poly(I:C) treated (**Figure 5H**). Above all, these results demonstrated that miR-15b directly targets TBK1 gene, and TBK1 expression could be regulated by miR-15b during RNA virus infection.

### miR-15b Regulates TBK1-Mediated Signaling

**Figure 6:**
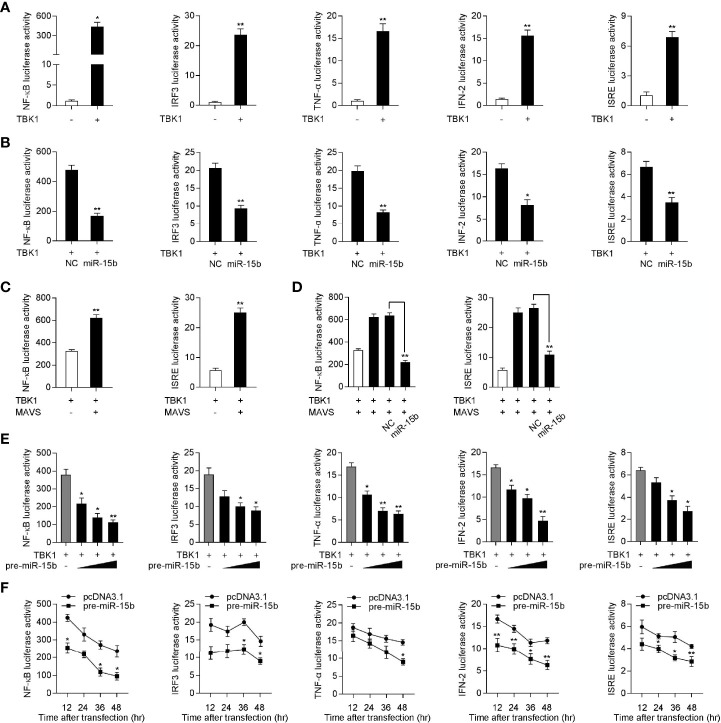
Overexpression of miR-15b inhibits TBK1-mediated NF-κB and IRF3 signaling. **(A)** The dual-luciferase reporter assayed the activation of a series of luciferase reporter genes during TBK1 gene transfection, including NF-κB, IRF3, TNF-α, IFN-2, and ISRE luciferase reporters. **(B)** The luciferase activity was tested the role of miR-15b on indicated luciferase reporters upon TBK1 expression plasmid transfection in EPC cells. **(C)** EPC cells were cotransfected with MAVS expression plasmid, together with TBK1 expression plasmid, and the NF-κB and IRF3 luciferase reporter genes for 48 h, then the luciferase activity was detected. **(D)** TBK1 expression plasmid, MAVS expression plasmid, NC or miR-15b, together with luciferase reporter gene was transfected into EPC cells for 48 h. Then, the luciferase activity was detected **(E)** The dual-luciferase reporter analysis was conducted to determine the role of pre-miR-15b on indicated luciferase reporters upon TBK1 expression plasmid transfection in EPC cells. **(F)** The time gradient experiment was conducted for pre-miR-15b transfection. All the luciferase activity was normalized to renilla luciferase activity. All data are presented as the means ± SE from three independent triplicated experiments. **p < 0.01; *p < 0.05 versus the controls.

Given that mammals TBK1 plays a crucial role in the innate immunity through inducing NF-κB and IRF3 signals, we want to determine the underlying regulatory mechanism of miiuy croaker miR-15b in the TBK1-induced antiviral pathway (33, 34). Similar to mammals, increasing evidence have shown that TBK1 in fish also participate in regulating IRF3-mediated IFN signaling pathway in response to virus infection (13). To measure the regulation function of miiuy croaker TBK1, we tested whether overexpression of TBK1 influences the activity of luciferase reporters, such as NF-κB, IRF3, TNF-α, IFN-2, and ISRE. The dual-luciferase reporter assays showed that miiuy croaker TBK1 could activate NF-κB and IRF3 luciferase reporter genes, as well as TNF-α, IFN-2, and ISRE luciferase reporter genes (**Figure 6A**). Previous results showed that miR-15b targets and regulates TBK1 expression, we then examined whether miR-15b regulates the activity of the indicated luciferase reporter genes. Subsequently, miiuy croaker TBK1 expression plasmid, together with miR-15b mimics or negative control mimics were transfected into EPC cells. The results were shown in **Figures 6B, D**, compared with negative control mimics, miR-15b mimics significantly impaired the activation of NF-κB, IRF3, TNF-α, IFN-2, and ISRE under transfection of TBK1 expression plasmid. Meanwhile, we tried to determine whether miiuy croaker pre-miR-15b could regulate the activity of the indicated luciferase reporter genes. We investigated that the downregulation produced by pre-miR-15b was presented a dose-dependent manner (**Figure 6E**). Then miiuy croaker TBK1 expression plasmid and pre-miR-15b plasmid were cotransfected into EPC cells, and that the results showed that pre-miR-15b exerts a decrease role in the activation of the indicated luciferase reporter genes from 12 h to 48 h post-transfection (**Figure 6F**). In short, these data revealed that miiuy croaker TBK1 could enhance NF-κB and IRF3 signaling, and miR-15b negatively regulates TBK1-mediated NF-κB and IRF3 signaling in a manner dependent on inhibiting TBK1.

### Knockdown of TBK1 Inhibits RNA Virus-Triggered Antiviral Responses

**Figure 7:**
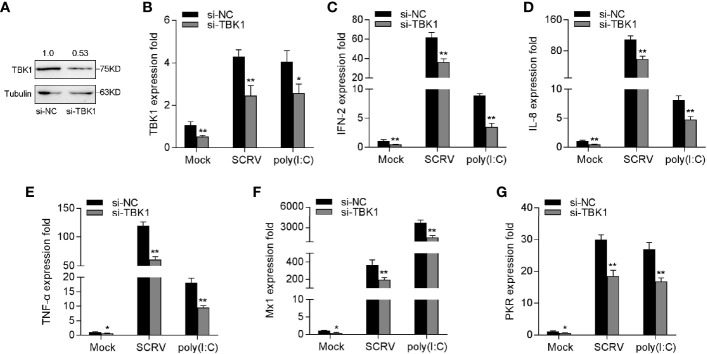
Knockdown of TBK1 induces an attenuation on the IFN-2 and antiviral genes expression. **(A, B)** TBK1 expression was detected by western blotting or qRT-PCR in MKC cells with control siRNA (si-NC) or TBK1 siRNA (si-TBK1) treatment for 48 h, and then infected with SCRV for 24 h. **(C–G)** MKC cells were transfected with si-NC or si-TBK1 for 48 h, and infected with SCRV for 24 h. The expression levels IFN-2 **(C)**, IL-8 **(D)**, TNF-α **(E)**, Mx1 **(F)** and PKR **(G)** were determined and normalized to GAPDH. All data are presented as the means ± SE from three independent triplicated experiments. **p < 0.01; *p < 0.05 versus the controls.

To verify the role of TBK1 in the antiviral immune response, we tested the expression of antiviral genes and inflammatory cytokines under SCRV or poly(I:C) treatment by silencing TBK1. As shown in **Figures 7A, B**, TBK1-specific siRNA effectually inhibited the level of TBK1. Afterwards, MKC cells were transfected with TBK1-specific siRNA and then treated with SCRV or poly(I:C), respectively. As can be seen from **Figures 7C–G**, knockdown of TBK1 observably weakened the levels of IFN-2, IL-8, TNF-α, Mx1, and PKR in MKC cells, which created an inhibition effect like miR-15b overexpression. Above of all results confirmed that miR-15b plays a regulatory role in host antiviral immunity through inhibition of endogenous TBK1, thereby inhibiting the production of antiviral genes and inflammatory cytokines.

### miR-15b Feedback Enhances SCRV Replication

**Figure 8:**
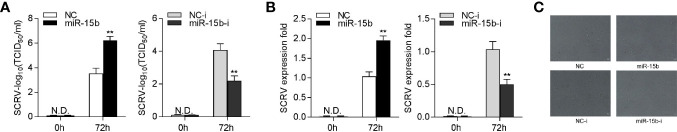
miR-15b promotes SCRV replication. **(A)** TCID_50_ assay of the virus replication. MKC cells were transfected with NC, miR-15b, NC-i, or miR-15b-i and infected with SCRV at MOI 5 for 1 h and washed and then added with fresh medium. After 72 h, SCRV TCID_50_ in cultural supernatants was measured with EPC cells. **(B)** MKC cells were transfected with oligonucleotides and then infected with SCRV, then the qRT-PCR analysis was conducted for intracellular virus RNA. **(C)** MKC cells were transfected with NC, miR-15b, NC-i, or miR-15b-i and infected with SCRV. Then, cells were observed for morphological changes. All data are presented as the means ± SE from at least three independent triplicated experiments. **p < 0.01 versus the controls.

To research the biological significance of intracellular miR-15b upregulation upon SCRV infection, we explored the function of miR-15b on SCRV replication in EPC cells. Through detecting the SCRV Tissue Culture Infection Dose 50% (TCID_50_) levels in the supernatant from the infected MKC cells, we found that the miR-15b contributes greatly to the replication of SCRV, while miR-15b inhibitors suppresses SCRV replication (**Figure 8A**). Consistent with above results, miR-15b mimics promoted SCRV replication, whereas inhibition of miR-15b weakened SCRV replication in the intracellular from the infected MKC cells (**Figure 8B**). Furthermore, a dramatically cytopathic effect (CPE) was observed in MKC cells after transfecting with miR-15b mimics (**Figure 8C**). These data indicated that miiuy croaker miR-15b has the ability to increase SCRV replication.

### miR-15b Regulation of TBK1 Is Ubiquitous in Teleost Fish

**Figure 9:**
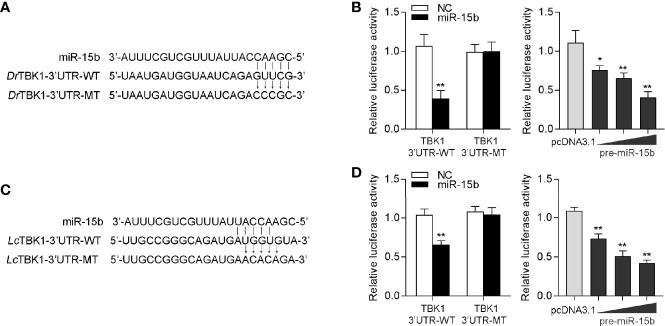
miR-15b-regulating TBK1 gene has been found in other teleosts. **(A)** Diagrammatic drawing of the predicted target sites of miR-15b in 3′-UTR of *D. rerio* TBK1. **(B)** Transfection of NC or miR-15b, along with the *D. rerio* TBK1 3′-UTR WT or the *D. rerio* TBK1 3′-UTR MT into EPC cells for 48 h and the luciferase activity was detected (left panel). The concentrate gradient experiment was conducted for pre-miR-15b transfection (right panel). **(C)** Diagrammatic drawing of the putative target sites of miR-15b in *L. crocea* TBK1 3′-UTR. **(D)** Transfection of NC or miR-15b, along with the *L. crocea* TBK1 3′-UTR WT or the *L. crocea* TBK1 3′-UTR MT for 48 h and the luciferase activity was examined (left panel). The concentrate gradient experiment was conducted for pre-miR-15b transfection (right panel). All the luciferase activity was normalized to renilla luciferase activity. All data are presented as the means ± SE from at least three independent triplicated experiments. **p < 0.01; *p < 0.05 versus the controls.

**Figure 10:**
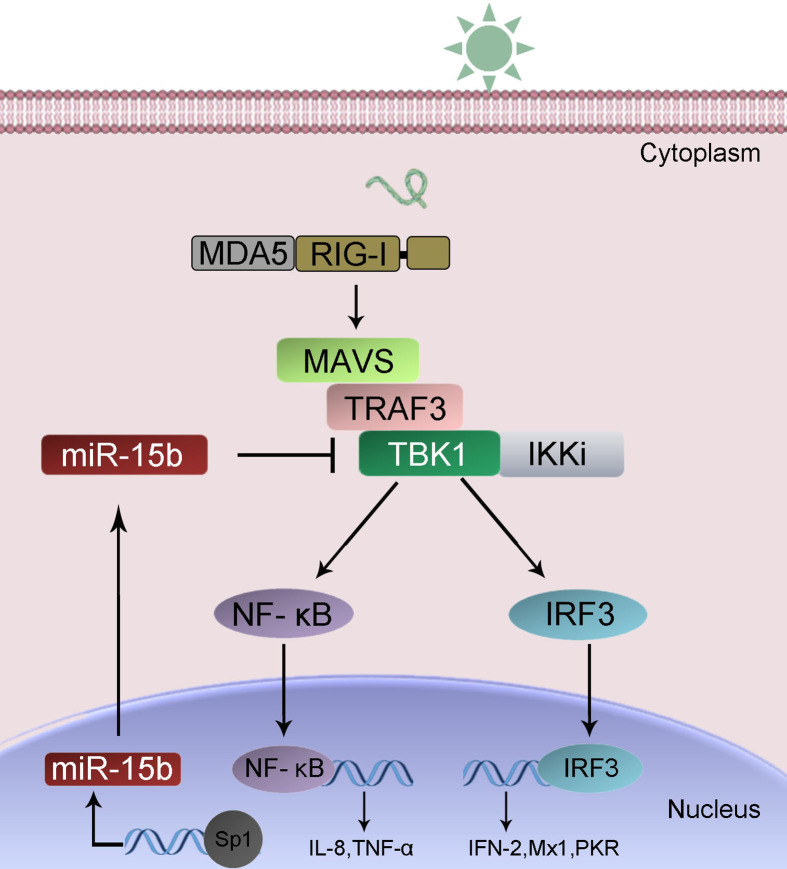
A proposed model for the mechanism by which induced miR-15b negatively regulates Type I IFN and inflammatory cytokine production in a manner by targeting TBK1 and inhibiting NF-κB and IRF3 signaling.

To confirm the universality of our investigation results that miR-15b regulates TBK1, we explored the findings in other teleost fish, such as zebrafish (*Danio rerio*) and large yellow croaker (*Larimichthys crocea*). Therefore, we constructed the luciferase reporters by cloning *D. rerio* TBK1 3′-UTR into pmir-GLO vector within the mutational binding site of miR-15b as a control (**Figure 9A**). The results can be seen in the dual-luciferase reporter assays, miR-15b mimics decreased the luciferase activity significantly when cotransfected with the *D. rerio* TBK1 3′-UTR reporter plasmid, while miR-15b mimics exerts no influence on the luciferase activity when cotransfected with mutant *D. rerio* TBK1 3′-UTR reporter (**Figure 9B**, left panel). What is more, pre-miR-15b plasmid also could inhibit the luciferase activity of TBK1 3′UTR and display a dose-dependent effect (**Figure 9B**, right panel). Meanwhile, we found that miR-15b presented a similar effect on the inhibition of luciferase activity when cotransfected with the *L. crocea* TBK1 3′-UTR (**Figures 9C, D**). These data showed that the findings that miR-15b targeting TBK1 gene also exists in other teleost fish, which verify that the functions of miR-15b are conserved to some extent. In total, these results indicated that miR-15b expression could be significantly enhanced by RNA virus, such as SCRV. Upregulated miR-15b inhibits type I IFN, ISGs and inflammatory cytokine production *via* targeting TBK1 and subsequently suppressing NF-κB and IRF3 antiviral signaling, thus facilitating viral replication (**Figure 10**).

## Discussion

Evidence is rapidly growing that miRNA plays vital roles in the generating process of immune responses and inflammation. In the past ten years, the studies of miRNA-mediated regulation mechanism have been improved constantly in teleost fish, which broaden our understanding of non-coding genes in fish group. Until now, although a wide variety of fish miRNA has been identified, its number presenting in fish is still on the rise. However, it still needs to make great efforts to enhance the understanding of the complex miRNA regulation mechanism existing in fish in regulating innate immunity. In this article, the underlying mechanisms inducted by miRNA to avoid immoderate production of type I IFN upon RNA virus infection were addressed in teleost fish. miR-15b has been certified to play a negatively regulatory role involved in antiviral innate immune responses in miiuy croaker. Concretely, host miR-15b could be induced upon SCRV infection. Enhanced miR-15b inhibits the production of antiviral genes and inflammatory cytokines *via* targeting TBK1, thus promoting the massive replication of the virus. Furthermore, we found that transcription factor Sp1 could activate miR-15b transcription. These findings reveal a new mechanism of action between the virus and the host, which contributes to the virus escape. The above results provide a theoretical basis for further enriching the miRNA-regulated immune response network in teleost fish.

Upon viral infection, structurally conserved viral components are recognized by PRRs that induces a series of signaling events to produce type I IFN and proinflammatory cytokines, which are vital to clear the invading virus. Type I IFN production play essential roles in host antiviral innate immune response, but the excessive production of type I IFN could lead to the situation of immunopathological (35). Therefore, to maintain host immune balance under virus infection, the production of type I IFN should be closely controlled by initiating appropriate antiviral response (35, 36). For instance, RNF125, IFI35, NLRC5, USP25, and A20 have been shown to negatively regulate RIG-I-induced antiviral signaling upon pathogen infection (37). Although the host produces sorts of regulatory mechanisms to eliminate viral invasion, virus has also evolved a variety of evasion strategies to achieve persistent infection, including specifically disrupting type I IFN production and its downstream molecules. Dengue virus NS2A and NS4B proteins could inhibit the induction of IFN-β by blocking TBK1/IRF3 phosphorylation (11). Ebola virus VP35 protein has been reported to prevent the phosphorylation, dimerization, and nuclear translocation of IRF3 induced by virus infection, thereby inhibiting the expression of type I IFN (38). In this study, we explored that host miR-15b as a critical regulator represses the production of SCRV-triggered type I IFN in miiuy croaker by inhibiting TBK1-mediated signaling pathways, thereby facilitating viral replication.

TBK1 functions as pivotal components of the virus-activated kinase complex that subsequently phosphorylate and activate IRF3 to induce type I IFN production. A growing number of studies has shown that TBK1 activity can be regulated by various different molecules. For instance, glycogen synthase kinase 3β physiologically binds to TBK1, facilitates TBK1 auto-phosphorylation at Ser172, leading to its kinase activation loop and activating TBK1 upon viral infection (39). It has been found that Siglec1 induces the degradation of TBK1 by means of the ubiquitin ligase TRIM27, thereby inhibiting the antiviral innate immune response (40). SHP-2 could phosphatase inhibit TBK1 activity and thus negatively regulate type I interferon and proinflammatory cytokine production (41). In addition, studies also revealed that dozens of molecules, such as ISG56, ERRα, FOSL1, and DOK3, prevent the formation of functional TBK1-containing complexes to negatively regulated TBK1 activity (15). In teleost, several of different molecules, such as Nrdp1, SIKE, and MVP, have been shown to inhibit TBK1activity and thus suppress TBK1-mediated antiviral signaling (42–44). Although many studies have focused on protein regulators, extensive investigations have indicated that noncoding miRNAs function as important controllers involved in innate immune responses. Until now, decades of mammalian miRNAs have been demonstrated to directly regulate TBK1 activity. In mycobacterium bovis infected cells, host miR-199a suppresses cellular autophagy and decreases IFN-β level *via* targeting TBK1 (45). miR-19a could mediate the negative regulation of the NF-κB signaling pathway in endometritis by targeting TBK1 (46). However, miRNA-driven regulation of TBK1-mediated immune response in aquatic animals is very little known. Herein, we firstly demonstrated that host miRNA, miR-15b inhibits the production of type I IFN by targeting TBK1 to promote viral replication in teleost fish. Moreover, we investigated transcription factors in the transcriptional regulation process of miR-15b, and found that miR-15b expression could be activated by transcription factor Sp1 through binding to its promoter region.

All in all, this study presents that miR-15b plays a significant role in SCRV-induced antiviral response, and indicates that miR-15b acts as a negative regulator in modulating the production of ISGs, type I IFN and inflammatory cytokines induced by SCRV infection. TBK1 gene is discovered as a new target for miR-15b, which could mediate downstream NF-κB and IRF3 signaling. Furthermore, miR-15b targets TBK1 that were also confirmed in other model fishes, which indicated the conservatism of the non-coding RNA miR-15b in evolution. In general, these findings deepen the understanding of miR-15b functions, and also enrich the complex regulatory network of host-virus interactions that miRNAs involved.

## Data Availability Statement

The original contributions presented in the study are included in the article/**Supplementary Material**. Further inquiries can be directed to the corresponding author.

## Ethics Statement

The animal study was reviewed and approved by Research Ethics Committee of Shanghai Ocean University.

## Author Contributions

Conceived and designed the experiments: TX. Performed the experiments: RC, QC, WZ, TX. Analyzed the data: RC, QC, WZ. Contributed reagents/materials/analysis tools: RC, QC, WZ, TX. Wrote the paper: RC, QC, TX. All authors contributed to the article and approved the submitted version.

## Funding

This study was supported by National Key Research and Development Project (2018YFD0900503), Open Fund of CAS Key Laboratory of Experimental Marine Biology, Institute of Oceanology, Chinese Academy of Sciences (KF2019NO1) and National Natural Science Foundation of China (31822057).

## Conflict of Interest

The authors declare that the research was conducted in the absence of any commercial or financial relationships that could be construed as a potential conflict of interest.

## Supplementary Material

Supplementary Table 1 PCR primer information in this study.

Supplementary Table 2 The putative miR-15b targets.
