# Supplementary material for: The Sp1-Responsive microRNA-15b Negatively Regulates Rhabdovirus-Triggered Innate Immune Responses in Lower Vertebrates by Targeting TBK1
Source: Front Immunol. 2021 Jan 27;11:625828. doi: 10.3389/fimmu.2020.625828 (PMC7873567; doi:10.3389/fimmu.2020.625828)
Supplement: Supplementary Table 1 — PCR primer information in this study. [file DataSheet_1.pdf]

Supplementary Table 1 PCR primer information in this study

**Primer name**	**Sequences (5'-3')**
TBK1-qRT-F	AGAGCACCACCAACTACCT
TBK1-qRT-R	ACTTTGACGGCATACAGG
Mx1-qRT-F	GCTGCTTGTTTACTCCCA
Mx1-qRT-R	ACCTGCATCATCTCCCTC
PKR- qRT-F	GATGGAGAGAACAGGGGA
PKR- qRT-R	CTTGGGGAAAAGTTAGGG
TNF-α-qRT-F	GTTTGCTTGGTACTGGAATGG
TNF-α-qRT-R	TGTGGGATGATGATCTGGTTG
IL-8-qRT-F	AGCAGCAGAGTCTTCGT
IL-8-qRT-F	TCTTCGCAGTGGGAGTT
IFN-2-qRT-F	GCTCTGCCTTCCCTGCTA
IFN-2-qRT-F	CAGTTGACTCCGCCCTCT
SCRV-qRT-F	GGGCTGGATGATAGACGATTG
SCRV-qRT-F	TGGCGGAGGTGCTTGATATGG
miR-15b-2-qRT-F	GCAGCGAACCATTATTTGC
miR-15b-2-qRT-R	TCCAGTTTTTTTTTTTTTTTAAAGCAG
5.8S-RT-F	AACTCTTAGCGGTGGATCA
5.8S-RT-R	GTTTTTTTTTTTTTTTGCCGAGTG
GAPDH-qRT-F	ACCTTCACTCCTCCATCTT
GAPDH-qRT-R	AGGTCACAGACACGGTTG
pre-miR-15b-2-HindIII-F	CCCAAGCTTAGATTTCCTGCTCTTCGT
pre-miR-15b-2-EcoRI-R	CCGGAATTCTGGGGTCTATTCTCACAC
TBK1-3'UTR-WT-NheI-F	CTAGCTAGCAAGGAGGAGATGGAGGGAGT
TBK1-3'UTR-WT-XbaI-R	TGCTCTAGACCGGCAACAATGAAGTGAGT
TBK1-3'UTR-MT-F	TATCGGCATCCACTCCATACGGTCTGCTTGGTCC
TBK1-3'UTR-MT-R	ATGGAGTGGATGCCGATACAAAAAGCTTCATCTCACAGTCG
TBK1-KpnI-F	GACGACAAGAAGCTTGGTACCATGCAGAGCACCACCAACTACC
TBK1-XbaI-R	TATAGAATAGGGCCCTCTAGACGGCAACAATGAAGTGAGTAACA
Sp1-KpnI-1F	CGGGGTACCATGAGCAATCAGCAGCAGG
Sp1-EcoRI-1R	CCGGAATTCACATCCATCAAGGCCGTCT
GFP-TBK1-3'UTR-F	CGGGGTACCGCTAGAAGGAGGAGATGGAGGGAGT
GFP-TBK1-3'UTR-R	CGCGGATCCCCGGCAACAATGAAGTGAGT
GFP-TBK1-3'UTR-MT-F	TATCGGCATCCACTCCATACGGTCTGCTTGGTCC
GFP-TBK1-3'UTR-MT-R	ATGGAGTGGATGCCGATACAAAAAGCTTCATCTCACAGTCG
TSS-2417-F	CGAGCTCTTACGCGTGCTAGCATGAAATTGAATGAGATGATATTATTGTTT
TSS-2025-F	CGAGCTCTTACGCGTGCTAGCATCTTGTTACTTACATAGCATAATAAAATAAAAT
TSS-1303-F	CGAGCTCTTACGCGTGCTAGCATAGTGTTGTAGTGTAGGGTCATCTAAGAC
TSS-979-F	CGAGCTCTTACGCGTGCTAGCTGAGATAGGCTGACTTAGAATAATAATATTG
TSS-427-F	CGAGCTCTTACGCGTGCTAGCTCAGAAGAGGTGGGAGTGTAACTAGT
TSS-Universal R	ACTTAGATCGCAGATCTCGAGCAAATCTTTTGAAGGGGTTGACC

Sp1 mut1 F	TCCCCCGGGAGTAGAAGGGGGACGAGAG
Sp1 mut1 R	TCCCCCGGGTTCTTTCATACATAAAAGT
Sp1 mut2 F	TCCCCCGGGAGGCCTGAAAAAGTGACCTTG
Sp1 mut2 R	TCCCCCGGGTTCTACTGGCTTCTCCTTTCT
Sp1 mut3 F	TCCCCCGGGATAGTCATGCAACTATGTCAG
Sp1 mut3 R	TCCCCCGGGTTTAGCTTGTTCTCCAAGGTC
Sp1 mut4 F	TCCCCCGGGAAACACACACACACACACAAA
Sp1 mut4 R	TCCCCCGGGACTCTCCCTGGATGTTAGA
Sp1 mut5 F	TCCCCCGGGAACCAAGCATTCACCTGAAAC
Sp1 mut5 R	TCCCCCGGGACTGTGGCTAGTGGTGGTTTT
*Dr*TBK1-3’UTR-WT-NheI F	TGTTTAAACGAGCTCGCTAGCACACACAGTCTCTGTCTGTCTCTTGTAC
*Dr*TBK1-3’UTR-WT-XhoI R	CAGGTCGACTCTAGACTCGAGGAACTCGGACTTTACAAACTGCATT
*Dr*TBK1-3’UTR-MT-F	ATGGTAATCAGACCCGCCCACAGTGCAATGAACCACCA
*Dr*TBK1-3’UTR-MT-R	GCGGGTCTGATTACCATCATTACTAACTCCAGC
*Lc*TBK1-3’UTR-WT-NheI F	TGTTTAAACGAGCTCGCTAGCCAGTTTAATTAGATTATTTAACTTTGACGTT
*Lc*TBK1-3’UTR-WT-XhoI R	CAGGTCGACTCTAGACTCGAGTAACTCAGTTATTGATTATTGATCGGC
*Lc*TBK1-3’UTR-MT-F	TGTAACTGCTGTCATGGGGAAATGACATTTGTC
*Lc*TBK1-3’UTR-MT-R	CCCATGACAGCAGTTACAAATTACCTTCACGAGTTTATTCATGTTA
