# Supplementary material for: The Sp1-Responsive microRNA-15b Negatively Regulates Rhabdovirus-Triggered Innate Immune Responses in Lower Vertebrates by Targeting TBK1
Source: Front Immunol. 2021 Jan 27;11:625828. doi: 10.3389/fimmu.2020.625828 (PMC7873567; doi:10.3389/fimmu.2020.625828)
Supplement: Supplementary Table 2 — The putative miR-15b targets. [file DataSheet_2.pdf]

Supplementary Table 2 The putative miR-15b targets

miRNA	Putative target genes
miR-15b	TANK-Binding Kinase 1 (TBK1)
	Activating Transcription Factor-2
	calcium voltage-gated channel subunit alpha1 E
	Casitas B-lineage lymphoma
	cAMP responsive element binding protein 3
	colony stimulating factor 1 receptor
	eukaryotic translation initiation factor 4E
	fibronectin 1
	hepatocyte growth factor
	interleukin 10 receptor subunit beta
	interleukin 1 receptor accessory protein
	interleukin 7 receptor
	leukemia inhibitory factor receptor
	mannan-binding lectin serine protease 1
	nuclear factor of activated T cells 1
	Nerve growth factor receptor
	paxillin
	transforming growth factor beta 3
	thrombomodulin
	tyrosine kinase with immunoglobulin like and EGF like domains 1
	TNF receptor superfamily member 21
	Protein phosphatase 2A regulatory subunit 3
	Protein phosphatase 2A regulatory subunit 1
	Fas ligand
